# Markedly Discordant and Rapidly Changing Hemoglobin A1c Values in a Normoglycemic Patient: A Case Report

**DOI:** 10.7759/cureus.108291

**Published:** 2026-05-05

**Authors:** Osama Elkhider, Omer Elshaikh, Alisha Sharma

**Affiliations:** 1 Internal Medicine, Medical College of Wisconsin, Milwaukee, USA; 2 Pathology and Laboratory Medicine, NEOM Hospital Operated by Fakeeh Care, Sharma, SAU; 3 Internal Medicine, VCU Medical Center North Hospital, Richmond, USA

**Keywords:** assay interference, discordant hba1c, glycation kinetics, hemoglobin a1c (hba1c), opioid exposure

## Abstract

Hemoglobin A1c (HbA1c) reflects average glycemia over approximately two to three months and is widely used for the diagnosis and monitoring of diabetes mellitus; however, discordant results may occur due to analytical interference, altered erythrocyte turnover, or preanalytical factors. We report a 31-year-old male with no known history of diabetes who presented for routine laboratory evaluation and was found to have an HbA1c of 13.9% despite a normal fasting plasma glucose of 89 mg/dL. Repeat testing of the same sample six hours later demonstrated a decrease to 7.5%, and a simultaneously collected new sample yielded an identical value. Over subsequent days, repeated measurements across multiple samples showed further decline and eventual stabilization at 5.8%. The patient remained asymptomatic, and additional evaluation did not support persistent hyperglycemia. Urine toxicology screening was positive for opioids, and the patient reported recent use of an unidentified substance. A follow-up sample obtained after two weeks confirmed a stable HbA1c of 5.8%. The rapid intra-sample variability and subsequent normalization observed in this case are inconsistent with the known kinetics of hemoglobin glycation and strongly suggest a non-physiological mechanism, most likely assay interference. This case emphasizes the importance of verifying unexpected HbA1c results prior to clinical decision-making to avoid misdiagnosis and inappropriate management.

## Introduction

Hemoglobin A1c (HbA1c) is formed by nonenzymatic glycation of hemoglobin and reflects average glycemia over approximately two to three months [1,2]. Its interpretation assumes a stable erythrocyte lifespan and the absence of interfering factors.

Several conditions may affect HbA1c independently of glucose, including anemia, hemoglobin variants, chronic kidney disease, and liver disease [3-5]. Analytical interference related to assay methodology or exogenous substances has also been reported [5,6]. When HbA1c values are discordant with plasma glucose, further evaluation is required to avoid diagnostic error. In such cases, alternative markers of glycemia, including fructosamine, glycated albumin, or continuous glucose monitoring, may provide a more accurate assessment of glycemic status.

## Case presentation

A 31-year-old male with no significant past medical history presented for routine laboratory evaluation. He was asymptomatic and denied polyuria, polydipsia, weight loss, or fatigue. He was not taking any medications.

Physical examination was unremarkable. Vital signs were normal, and there were no signs of acute illness.

Initial laboratory testing revealed a fasting plasma glucose of 89 mg/dL and a markedly elevated HbA1c of 13.9% (sample A, 12:00 p.m.). Repeat testing of the same sample, approximately six hours later, showed a decrease to 7.5%. At the same time, a newly collected sample (sample B) also yielded an HbA1c of 7.5%, confirming the reproducibility of the revised measurement.

Over the following days, testing of both samples demonstrated further decline in HbA1c values, reaching 6.5% and then 5.8%. On day three, an additional newly collected sample (sample C) also showed an HbA1c of 5.8%. Continued repeated testing across all available samples demonstrated stable values of 5.8% thereafter (Table 1). At each time point, results were consistent across multiple samples, reducing the likelihood of random analytical error. Stored samples were maintained under standard laboratory conditions and reanalyzed to assess reproducibility. These rapid changes in HbA1c values over hours to days were inconsistent with expected glycation kinetics.

**Table 1:** Temporal Pattern of HbA1c Measurements Across Samples

Day	Sample	Time	HbA1c (%)	Interpretation
Day 0	Sample A	12:00	13.9	Initial discordant value
Day 0	Sample A (repeat)	18:00	7.5	Intra-sample variability
Day 0	Sample B	18:00	7.5	Inter-sample reproducibility
Days 1-2	Samples A and B	Not recorded	6.5	Converging values
Day 3	Samples A and B	Not recorded	5.8	Stabilization
Day 3	Sample C	Not recorded	5.8	New sample confirms value
Days 4-13	Samples A, B, and C	Not recorded	5.8	Persistent stability
Day 14	Sample D	Not recorded	5.8	New sample after two weeks without substance use

Upon further questioning, the patient reported recent use of an illicit substance but could not identify the specific agent. Urine toxicology screening was positive for opioids.

After two weeks, the patient returned for repeat testing. He reported no further use of illicit substances during this period. A newly collected sample (sample D) demonstrated an HbA1c value of 5.8%, consistent with prior measurements.

Additional laboratory evaluation to assess common causes of HbA1c discordance was unremarkable (Table 2). All samples were visually inspected and showed no evidence of hemolysis, lipemia, or icterus. Laboratory quality control data for the analyzer were within acceptable limits, and no analyzer flags were recorded.

**Table 2:** Laboratory Results

Laboratory Test	Results	Reference Range
Hemoglobin (g/dL)	14.1	13.7-17.5
Creatinine (mg/dL)	1.1	0.6-1.3
Aspartate aminotransferase (U/L)	18	<50
Alanine aminotransferase (U/L)	23	<41
Total bilirubin (mg/dL)	0.3	0.6-1.2
Serum glucose (mg/dL)	89	70-180
Urinalysis	Unremarkable (including negative glucose and ketones)	Normal

HbA1c measurements were performed using an immunoturbidimetric assay on a Roche Cobas c111 analyzer (Roche Diagnostics, Indianapolis, IN). All samples were processed under standard laboratory conditions, with minimal delay between collection and analysis. Samples were stored and handled according to institutional protocols, and no deviations in sample handling or storage conditions were identified that could account for the observed variability. The assay was performed using routine laboratory calibration and quality control procedures in accordance with manufacturer and institutional standards. Repeat testing at each time point was performed on multiple samples to verify reproducibility.

## Discussion

This case demonstrates a highly unusual pattern of HbA1c measurements characterized by marked intra-sample variability followed by convergence and stabilization across multiple independently collected samples.

The observed changes are biologically implausible. HbA1c reflects glycation over the approximately 120-day lifespan of erythrocytes [2,4]. A decrease from 13.9% to 7.5% within six hours would require near-complete replacement of circulating erythrocytes, which did not occur. Such rapid changes over hours to days are incompatible with known erythrocyte lifespan and glycation kinetics, making it unlikely that the measured values reflect true glycemic status.

The pattern of results further supports a non-physiological mechanism. The initial discrepancy within the same sample suggests measurement instability, while subsequent agreement across independently collected samples argues against random error and instead supports a systematic factor affecting the assay. Common causes of discordant HbA1c, including anemia, renal dysfunction, and liver disease, were excluded by normal laboratory findings [3,5], further supporting a non-physiological explanation.

The measurement principle of the assay used in this case is relevant when considering possible sources of interference. The Tina-quant immunoturbidimetric inhibition assay relies on a monoclonal antibody directed against the glycated N-terminal hexapeptide of the β-globin chain; antigen-antibody complexes agglutinate with a polyhapten reagent, and the resulting change in turbidity is measured photometrically against total hemoglobin to give a percent HbA1c value [6]. Because the readout depends on antibody recognition of a short surface peptide, the method can be affected by factors that chemically modify the β-chain N-terminus, generate circulating epitopes that compete with native HbA1c for antibody binding, or alter the optical signal itself [5,6]. These mechanisms provide a plausible explanation for the discordant and rapidly changing HbA1c values observed in this case.

Several drugs can cause discordant HbA1c results, either by altering erythrocyte survival, chemically modifying hemoglobin, or interfering directly with the assay [7]. Aspirin acetylates globin chains and can falsely raise HbA1c on charge-based methods [8-10]. Dapsone, ribavirin, and some antiretrovirals lower HbA1c by shortening red cell lifespan [7,11]. High doses of vitamin C have also been reported to alter HbA1c values, with the direction of bias depending on the assay used [9,12]. Opioid use has been associated with discordant HbA1c values in prior reports [13], although the underlying mechanism remains uncertain.

The temporal pattern observed is consistent with a transient factor affecting HbA1c measurement. Progressive normalization across multiple samples, along with stable results on a newly collected sample after two weeks without reported substance use, suggests that any potential interfering factor may have diminished or resolved. The fall from 13.9% to 7.5% over six hours, with a fresh sample drawn at the later time point showing an identical value, is most consistent with an interferent that was either decaying ex vivo or being cleared from the patient’s circulation. This behavior resembles, though does not precisely match, the labile A1c (pre-A1c) intermediate, an unstable Schiff base formed prior to Amadori rearrangement. While modern assays incorporate steps to minimize labile A1c interference, adducts derived from xenobiotics or their metabolites may not be fully eliminated. In this context, a drug- or metabolite-induced modification of the β-chain N-terminus could generate a cross-reactive epitope, with subsequent loss of the modifying group accounting for normalization [8,14,15]. However, in the absence of confirmatory testing using alternative analytical methods, the presence and nature of such interference cannot be definitively established.

Urine toxicology confirmed recent opioid exposure, and the patient reported substance use prior to the initial measurement. Although a temporal association exists, the specific agent was not identified, and a causal relationship cannot be established. It should also be noted that illicit opioid preparations frequently contain adulterants and contaminants, any of which could theoretically have acted as the responsible interferent rather than the opioid itself.

Consistent results across multiple samples at each time point argue against random analytical error but do not exclude systematic interference. The absence of confirmatory testing using alternative methodologies or independent glycemic markers (such as fructosamine or glycated albumin) limits further interpretation. Continuous glucose monitoring, where available, would also have allowed direct exclusion of true hyperglycemia. Nevertheless, this case highlights the importance of correlating HbA1c results with clinical findings and plasma glucose values and emphasizes that unexpected or rapidly changing HbA1c results should be verified before clinical decision-making [6,16].

## Conclusions

This case highlights a markedly discordant HbA1c pattern with rapid intra-sample variability and subsequent stabilization across multiple samples. The findings are incompatible with physiological glycation and likely represent a non-physiological mechanism. Verification of unexpected HbA1c results is essential to avoid misdiagnosis and inappropriate treatment of diabetes.
